# Impact of ultraviolet filters and polycyclic aromatic hydrocarbon from recreational activities on water reservoirs in southeast Queensland Australia

**DOI:** 10.1093/etojnl/vgaf007

**Published:** 2025-01-09

**Authors:** Rory Verhagen, Cameron Veal, Elissa O’Malley, Michael Gallen, Katrin Sturm, Michael Bartkow, Sarit Kaserzon

**Affiliations:** Queensland Alliance for Environmental Health Sciences (QAEHS), The University of Queensland, Woolloongabba, QLD, Australia; Seqwater, Ipswich, QLD, Australia; School of Civil Engineering, The University of Queensland, St Lucia, QLD, Australia; Australian Rivers Institute, School of Environment and Science, Griffith University, Southport, QLD, Australia; Queensland Alliance for Environmental Health Sciences (QAEHS), The University of Queensland, Woolloongabba, QLD, Australia; Seqwater, Ipswich, QLD, Australia; Seqwater, Ipswich, QLD, Australia; Queensland Alliance for Environmental Health Sciences (QAEHS), The University of Queensland, Woolloongabba, QLD, Australia; Queensland Public Health and Scientific Services Division, Queensland Health, Herston, QLD, Australia

**Keywords:** passive sampling, drinking water, boating, swimming

## Abstract

Water reservoirs and lakes are gaining popularity for recreation activities as populations increase and green spaces become in high demand. However, these activities may cause contamination to critical water resources. This study investigates the impact of recreational activities on the presence and concentration of polycyclic aromatic hydrocarbons (PAHs) and ultraviolet (UV) filters in drinking water reservoirs in Southeast Queensland, Australia. Polydimethylsiloxane passive samplers were used to monitor 14 lakes over a 3-year period, focusing on seasonal variations and the influence of recreational activities such as petrol-powered boating and swimming. A total of 15 PAHs and six UV filters were detected, with chrysene (97%) and octyl salicylate (34%) being the most prevalent PAH and UV filter, respectively. Polycyclic aromatic hydrocarbon levels were statistically significantly higher in lakes permitting petrol-powered boating, especially during summer (*p *= 0.005 to 0.05). Lake Maroon and Lake Moogerah were the only sites that showed significantly higher PAH levels in summer (3.9 ± 1.1 and 4.0 ± 1.2 ng L^−1^, respectively) than winter (1.6 ± 0.61 and 1.5 ± 0.84, respectively). Ultraviolet filters were generally detected in higher levels in lakes allowing swimming, with Lake Moogerah and Lake Sommerset measuring UV filter concentrations of 20 ± 4.1 and 20 ± 11 ng L^−1^ in summer, respectively. Other lakes that do not permit swimming, such as Lake Maroon and Lake Samsonvale, also exhibited elevated UV filter levels, suggesting illegal swimming. These findings highlight the complexity of PAH and UV filter presence, influenced by multiple factors including lake size, recreational activity type, and seasonal variations. The levels of individual PAHs and UV filters in this study were below established freshwater guidelines. However, when considering their bioaccumulation potential and mixture toxicity, mitigating the impact of these substances on our environment and the organisms within it should be of priority.

## Introduction

In recent decades, studies have shown that water systems are increasingly contaminated by chemicals from human activities (Akhtar et al., 2021; Li & Wu, 2019; Schwarzenbach et al., 2006). One of the main sources of water quality deterioration is synthetic chemical contaminants and priority organic pollutants from wastewater effluents, with additional contamination from urban, agricultural practices, and atmospheric deposition (Comber et al., 2022; Kolpin et al., 2004; Moss, 2008). Recreational use of drinking water lakes is another source of contamination. As populations grow and green spaces become increasingly scarce, drinking water lakes are becoming popular recreational spots. Governments face the challenge of balancing recreational use with water quality maintenance (Miller et al., 2006). Since 2018, more drinking water lakes in Queensland, Australia, have opened for recreation. Although this has social, economic, and well-being benefits, it also increases water treatment costs to manage potential contaminants. Understanding the impact of recreational activities on water quality is crucial, especially in light of climate change, water scarcity, and security issues. In 2004, a workshop by the Cooperative Research Centres for Water Quality and Treatment highlighted the need for more research on the impacts of recreational activities on water quality, particularly with regard to chemical exposure (Miller et al., 2006).

Southeast Queensland is a subtropical region of Australia, with hot summers and regularly occurring bushfires. Seqwater manages 26 dams, 48 weirs, and two borefields for drinking water and irrigation purposes. Seqwater allows recreation at 21 sites; however, sites that allow on-water recreation are limited: 17 allow nonpowered boating (i.e., kayaking and canoeing), 12 allow electric powered boating, eight allow petrol-powered boating, and eight allow swimming. In 2014, Seqwater began a catchment and drinking water quality micropollutant monitoring program, with a focus on improving the characterization and understanding of chemical pollutant (i.e., pesticides, personal care products and polycyclic aromatic hydrocarbon [PAH]) risk in drinking water catchment areas, through annual summer and winter sampling campaigns (Kaserzon et al., 2014).

Polycyclic aromatic hydrocarbons are known for their mutagenic, toxic, and carcinogenic properties (Boström et al., 2002; Lawal, 2017) and are found in both petrogenic sources (i.e., natural components of crude oil or coal) and pyrogenic sources (i.e., incomplete combustion of fossil fuels or organic matter). Although they can enter the environment via natural sources such as bushfires, pyrogenic PAHs primarily originate from anthropogenic sources, such as fuel, biomass, and coal combustion (Howsam & Jones, 1998; Mojiri et al., 2019). One anthropogenic source that is associated with recreational activities is the use of leisure motorized boats, which release PAHs from their exhaust fumes and/or fuel leaks (Mastran et al., 1994; Mosisch & Arthington, 1998; Wang et al., 2022).

Ultraviolet filters, chemicals that are used in sunscreens and personal care products, have received great attention due to their high-volume use and bio-accumulative properties (including benzophenone 3 and octinoxate; Colas-Ruiz et al., 2023; Pintado-Herrera et al., 2020). Some UV filters (including benzophenones, 3-benzylidene camphor, and octyl salicylate) are known for their endocrine-disrupting effects on fish (Kunz & Fent, 2006, 2009), and octinoxate is listed under the European Community Rolling Action Plan and included on the watch list of the European Water Framework Directive (Nagorka & Duffek, 2021). They can enter the aquatic environment through wastewater effluent and direct release during recreational activities. Although they are susceptible to photodegradation, they are considered pseudo-persistent due to their constant release into the environment (Colas-Ruiz et al., 2023; Fent et al., 2010; Giokas et al., 2007; Kroon et al., 2020; O'Malley et al., 2021).

Due to the low levels of both PAHs and UV filters in water, silicone rubber passive samplers are used to concentrate contaminants in situ and have proven effective at monitoring hydrophobic chemicals such as PAHs (Allan et al., 2009; Rusina et al., 2007, 2010a, 2010b) and UV filters (Pintado-Herrera et al., 2016, 2020; Verhagen et al., 2019).

This study aimed to investigate whether there are differences in the presence and signature of PAHs and UV filters in drinking water reservoirs open to recreational activities versus those that are not and whether certain PAHs and UV filters can be used as chemical markers for a specific type of recreational activity.

## Material and methods

A detailed description of the materials used, preparation of passive flow monitors (PFMs) and gas chromatography/high-resolution mass spectrometry analysis can be found in the supplementary material.

### Study design

Seasonal concentration profiles of PAHs (which may have originated from hydrocarbon-based motorized boat use) and UV filters (associated with recreational swimming and kayaking) in Southeast Queensland lakes were investigated. The full list of PAHs and UV filters (commonly used in sunscreen) can be found in Table 1.

**Table 1.** Polycyclic aromatic hydrocarbons and UV filters percent of detection and the range of concentrations above limits of reporting (*n*g L^−1^) for different types of on-water recreation.

PAH	Percent detection across sites with petrol boats (*n* = 83)	**Concentration range (minimum–maximum; ng L^−1^)**	Percent detection across sites without petrol boats (*n* = 66)	**Concentration range (minimum–maximum;** **ng L^−1^)**
**Acenaphthylene**	33% (*n* = 27)	0.048–0.34	23% (*n* = 15)	0.056–1.2
**Acenaphthene**	14% (*n* = 12)	0.045–0.14	22% (*n* = 14)	0.49–0.18
**Fluorene**	14% (*n* = 12)	0.24–0.37	17% (*n* = 11)	0.20–0.53
**Phenanthrene**	33% (*n* = 27)	0.099–1.10	35% (*n* = 23)	0.093–1.2
**Anthracene**	17% (*n* = 17)	0.021–0.13	6% (*n* = 4)	0.021–0.11
**Fluoranthene**	83% (*n* = 69)	0.054–2.0	64% (*n* = 42)	0.055–0.79
**Pyrene**	59% (*n* = 49)	0.092–1.5	40% (*n* = 26)	0.084–1.3
**Benzo (a) anthracene**	77% (*n* = 64)	0.0012–0.061	52% (*n* = 34)	0.00078–0.013
**Chrysene**	97% (*n* = 81)	0.0050–0.095	81% (*n* = 53)	0.0034–0.028
**Benzo (bjk) fluoranthene**	81% (*n* = 67)	0.00055–0.019	55% (*n* = 36)	0.00076–0.0078
**Benzo (e) pyrene**	54% (*n* = 45)	0.0012–0.014	18% (*n* = 12)	0.0011–0.0057
**Benzo (a) pyrene**	37% (*n* = 31)	0.00049–0.0081	17% (*n* = 11)	0.00049–0.0044
**Indeno (1,2,3-cd) pyrene**	35% (*n* = 29)	0.0012–0.014	8% (*n* = 5)	0.0016–0.0025
**Dibenzo (a, h) anthracene**	ND	ND	1% (*n* = 1)	0.0021
**Benzo (g, h, i) perylene**	55% (*n* = 46)	0.0007–0.018	17% (*n* = 11)	0.0008–0.012

**UV filter**	**Percent detection across sites permits swimming (*n* = 63)**	**Concentration range (minimum–maximum; ng L^−1^)**	**Percent detection across sites no-swimming (*n* = 85)**	**Concentration range (minimum–maximum; ng L^−1^)**

**Octyl salicylate**	34% (*n* = 22)	0.88–7.5	21% (*n* = 18)	1.2–7.6
**Homosalate**	14% (*n* = 8)	1.5–9.5	16% (*n* = 14)	2.2–7.9
**Octyl methoxycinnamate**	10% (*n* = 7)	0.14–0.29	9% (*n* = 8)	0.11–0.50
**Octocrylene**	18% (*n* = 13)	0.39–2.77	11% (*n* = 9)	0.47–0.96
**4-methylbenzylidene camphor**	25% (*n* = 16)	1.1–16	16% (*n* = 13)	2.1–22
**Oxybenzone**	7% (*n* = 5)	1.9–13	2% (*n* = 2)	6.1–6.7

*Note.* ND = no detection; PAH = polycyclic aromatic hydrocarbon.

Polydimethyl siloxane (PDMS) silicon rubber passive samplers were deployed in 14 lakes of subtropical Southeast Queensland twice a year (July–August in winter, January–February in summer) between 2014 and 2017, over a period of 28 days. The samplers were deployed at an approximate depth of 3 m in each lake. Despite the approximate same deployment depth, the exposure to UV light could vary due to differing light filtering conditions across the lakes. We measured turbidity in nephelometric turbidity units for each lake. Our analysis indicated that Lake Kurwongbah and Lake Macdonald (LMD) had significantly higher turbidity levels compared with other lakes (see online supplementary material Table 9). This variation in turbidity did not show any clear patterns affecting the overall results. The deployment of samplers was conducted in alignment with the drinking and catchment water quality micropollutant passive sampling procedure (Kaserzon et al., 2014). Polydimethyl siloxane strips were deployed in stainless steel cages to detect the presence of nonpolar organic chemicals, such as certain PAHs and UV filters. Passive flow monitors were co-deployed with the passive samplers at each site to estimate water flow conditions during sampler deployment. Similar to the performance reference compound approach used to correct for in situ flow velocity effects on chemical specific sampling rates, the PFM allows to correct for in situ flow conditions in the system. However, instead of a performance reference compound, the dissipation rate of the gypsum inside the PFM is calculated and applied to the sampling rate calculations of the PAHs and UV filters to obtain water concentrations that are flow corrected. More details of the approach are provided in O'Brien, 2009, and O'Brien et al., 2012.

This study only used data from lakes that are used for drinking water supply and allow various degrees of recreational use (Table 2), as well as from three reference sites where no recreation is allowed, including one site that functions as drought mitigation storage.

**Table 2.** Deployment site names, site code, lake water storage volume, and indication of on-water recreational activity level. SEQ = Southeast Queensland.

Site name	Site code	Volume	Types of on-water recreation			
		ML	Boating nonpowered	Boating electric powered	Boating fuel powered	Swimming
**SEQ-Lake MacDonald**	LMD-SP001	8,018	No	No	No	No
**SEQ-Lake Borumba**	BOD-SP001	46,000	Yes	Yes	Yes	No
**SEQ-Poona Dam**	POD-SP001	655	No	No	No	No
**SEQ-Lake Baroon**	BPD-SP001	61,000	Yes	Yes	No	Yes
**SEQ-Ewen Maddock Dam**	EMD-SP001	16,587	Yes	No	No	Yes
**SEQ- Lake Somerset**	SOD-SP010	379,849	Yes	Yes	Yes	Yes
**SEQ- Lake Somerset**	SOD-SP011	379,849	Yes	Yes	Yes	Yes
**SEQ- Lake Somerset**	SOD-SP001	379,849	Yes	Yes	Yes	Yes
**SEQ-Lake Wivenhoe**	WID-SP004	1,200,000	Yes	Yes	Yes	Yes
**SEQ-Lake Wivenhoe**	WID-SP001	1,200,000	Yes	Yes	Yes	Yes
**SEQ- Lake Samsonvale**	NOD-SP091	193,291	Yes	Yes	No	No
**SEQ- Lake Samsonvale**	NOD-SP001	193,291	Yes	Yes	No	No
**SEQ- Lake Kurwongbah**	LAK-SP001	8,706	Yes	No	Yes	No
**SEQ-Lake Samsonvale**	NOD-SP023	193,291	Yes	Yes	No	No
**SEQ-Lake Wyaralong**	WYD-SP001	102,884	Yes	Yes	No	No
**SEQ-Lake Moogerah**	MOD-SP027	83,765	Yes	Yes	Yes	Yes
**SEQ-Lake Maroon**	MAD-SP004	44,319	Yes	Yes	Yes	No
**SEQ-Little Nerang Dam**	LND-SP014	6,705	No	No	No	No
**SEQ- Lake Advancetown**	HID-SP001	309,700	Yes	Yes	No	No
**SEQ- Lake Advancetown**	HID-SP002	309,700	Yes	Yes	No	No

### Preparation and extraction of PDMS

#### Preparation of PDMS sheets and sampling cages

Polydimethyl siloxane passive samplers were deployed in stainless steel cages. Before deployment, the PDMS strips (500 μm thick, 2.5 cm wide, 92 cm long) were cleaned with acetone for two consecutive 24-hr periods followed by n-hexane for two consecutive 24-hr periods to remove oligomers (Rusina et al., 2007). The sheets were then air dried and placed into clean glass jars until use.

For each site, one PDMS sheet was placed into the cage and kept in place with cable ties at each end. At some sites, PDMSs were deployed in duplicate. After assembling, the cages were placed into a metal can and stored at 4 °C prior to being transported to the sampling sites. Field sampling guides include the use of gloves for all sampler handling to avoid contamination. On retrieval, samplers were stored at 4 °C until extracted. The stability of UV filters during storage at 4 °C is not well understood. Although our samplers are stored in the dark and wrapped in aluminum foil to prevent UV exposure, further investigation is needed to assess potential degradation and its impact on concentration measurements.

#### Extraction of the PDMS sheets

Before extraction, the surface of the PDMS sheets was cleaned by rinsing with water and dried with KimWipes. In addition, PDMS sheets were spiked with 20 μl of isotope labelled PAH standards, d10-fluoranthracene and d12-chrysene (internal standards); these were also added to a not extracted side spike vial, which is used to calculate analyte recoveries during the extraction process. After this, each PDMS sheet was separately placed into a jar filled with 200 ml of n-hexane at room temperature (21 °C) and placed on a shaker (150 rpm) for two consecutive 24-hr periods, where the hexane was replaced in between the 24-hr periods. Aluminum foil was used to cover the jars to prevent light degradation of the extract. After the 48 hr of extraction, extracts were combined and reduced in volume by rotary evaporation. The extracts were then filtered using sodium sulphate columns to remove any water and then reduced to 0.5 ml under a gentle stream of nitrogen. The extracts were then filtered, using 0.45 μm PTFE filters, into a 2 ml amber glass vial. Extracts were then concentrated and adjusted to a final volume of 200 μl in hexane before analysis. At each sample and the side spike, 25 μl of ^13^C_12_PCB-141 (2 ng μl^−1^) was added as a recovery standard prior to analysis. Polydimethyl siloxane strips from replicate sites were extracted and analyzed individually.

### Data modeling

The uptake of a chemical into the passive sampler is initially a linear process, which eventually leads to equilibrium in the sampler (Vrana et al., 2005). The different characteristics of a chemical, such as its octanol-water partition coefficient *K*_ow_, and molecular weight, and environmental factors, such as temperature, turbulence, and flow, can affect the uptake rate of chemicals (Huckins et al., 2006). To estimate water concentrations (C_*w*_), when the chemical uptake is in linear phase, the following equation can be applied:


(1) $$C_{w}=\frac{C_{s}M_{s}}{R_{s}t}=\;\frac{N_{s}}{R_{st}}$$

When equilibrium is reached, uptake is described by


(2) $$C_{w}=\frac{C_{s}}{K_{pw}}$$

where *C*_w_ is the concentration of the compound in water (ng L^−1^), *C*_S_ the concentration of the compound in the sampler (ng g^−1^), *M*_s_ the mass of the sampler (g), *N*_s_ the amount of compound accumulated by the sampler (ng), *R*_s_ the sampling rate (L day^−1^), *t* the deployment time (days), and *K*_pw_ the polymer-water partition coefficient (L g^−1^).

### Quality assurance/quality control

Unexposed PDMS sheets (laboratory blanks), and field blanks were included in every extraction batch. For PAHs, the analytical limit of detection (LOD) and quantification (LOQ) were previously calculated as three and 10 times the SD from seven spiked PDMS sheets, respectively (unpublished work, included in the online supplementary material tables). Levels of the blank were calculated as the mean of the blank plus three times the SD. The highest value (either the LOD or levels of the blank) is set as the limit of reporting (LOR). Mass in the samplers that fall between LOR and LOQ are labeled in online supplementary material Tables 1–6 with an asterisk. Similarly, UV filter LODs are included in online supplementary material Tables 1–6. Due to the ubiquitous nature of UV filters and their high background level, all of them were detected in similar levels in the laboratory and field blank samples, which resulted in high LOQs. The PAH internal standard recoveries ranged from 28% to 60% for d10-Ace and d12-I123cdp, respectively. Extraction recoveries for UV filters and internal standards for the silicone used in this study followed the procedures of Verhagen et al. (2019). The extraction recoveries for native UV filters ranged between 92% and 133% for BP8 and 3BC, respectively.

### Statistical analysis

Before statistical analysis, values below LOR were replaced with half the LOR (Hites, 2019). These adjusted values were included in the calculations of the sum and average concentrations of PAHs and UV filters. Statistical analyses were performed using GraphPad Prism 9.1.0. Initially, data normality was assessed by Shapiro-Wilk test. For normally distributed data, a one-way analysis of variance followed by Tukey’s multiple comparison test or unpaired *t* test was applied. For nonnormally distributed data, the Kruskal-Wallis test and Dunn’s multiple comparison test were used. Significance was set at *p *< 0.05.

## Results and discussion

### PAH detection frequency and chemical concentrations

**Figure 1.**
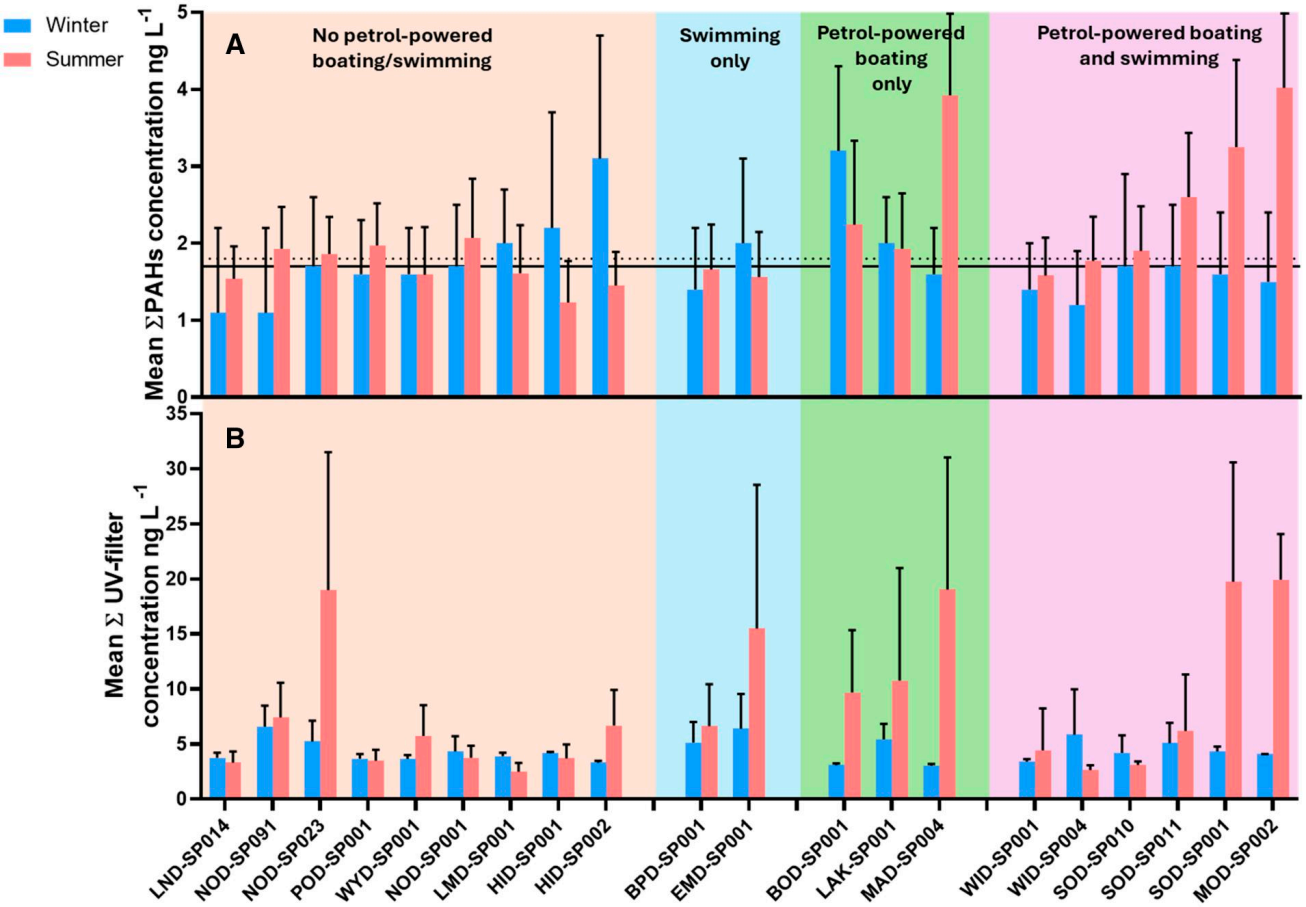
Average total polycyclic aromatic hydrocarbons (PAHs,A) and UV filter (B) concentration ± SD (ng L^-1^) over summer and winter sampling periods for seasons from winter 2014 to summer 2017 grouped by level of recreation. Solid line in Figure 1B is baseline whereas the dotted line is the burn input from control fires.

Although this study assessed the effect of petrol-powered boating on water quality, other anthropogenic activities also contribute to PAH levels. We established two baseline scenarios: one for nonspecific PAH contributions (average summer data for nonboating sites, referred to as Baseline in Figure 1) and one for PAH input from biomass reduction–controlled burns (average winter data for nonboating sites, referred to as Burn-input in Figure 1).

A total of sixteen PAHs were detected in the passive samplers. Due to the high levels of naphthalene detected in blank samples, it was not included in the study. Detection frequency ranged from 1% to 97% for dibenzo (a, h) anthracene and chrysene, respectively (Table 2).

At petrol-powered boating sites, chrysene had the highest detection frequency (97%), followed by fluoranthene (83%) and benzo(bjk)fluoranthene (81%). The lowest frequencies were for anthracene (17%), acenaphthene (14%), and fluorene (14%). At nonboating sites, chrysene (81%) was highest, followed by fluoranthene (64%) and benzo(bjk)fluoranthene (55%). Lower detection rates of two- and three-ringed PAHs in summer compared with winter could be due to their volatility, with water temperature differences ranging from 7 to 11 °C at Lake Baroon and Lake Moogerah.

Polycyclic aromatic hydrocarbon concentrations in winter and summer are listed in Table 1 and online supplementary material Table 1. Average ΣPAHs concentration at sites without petrol-powered boats ranged from 1.1 (± 1.1) to 3.1 (± 1.6) ng L^−1^ in winter and from 1.2 (± 0.54) to 2.1 (± 0.77) ng L^−1^ in summer (Figure 1). At sites with petrol-powered boats, average ΣPAHs ranged from 1.2 (± 0.7) to 3.2 (± 1.1) ng L^−1^ in winter and from 1.6 (± 0.49) to 4.0 (± 1.2) ng L^−1^ in summer (Figure 1). Mastran et al. (1994) detected 11 PAHs in a drinking water reservoir in Washington, USA, with concentrations during peak boating activity higher compared with our study of up to 2.1 µg/L and no PAHs detected during low boating activity, concluding high-speed boating as a major source of PAHs. Egardt et al. 2018 used passive samplers and found similar PAH levels (<4 ng/L) in Sweden’s marine environment to those found in our study. They observed significant seasonal changes, with heavy PAHs (5–6 rings) detected only during the peak season (July/summer), suggesting the impact of petrol-powered boating. A review reported PAH concentrations ranging from 0.03 to 8,300,000 ng L^−1^, with many studies showing higher values than this study (Mojiri et al., 2019).

For UV filters, high blank levels resulted in high LOQs, causing many results to fall below LOQs. Six UV filters were detected, with detection frequencies from 2% for oxybenzone to 34% for octyl salicylate (Table 2). Concentrations ranged from 0.11 to 22 ng L^−1^ for octyl methoxycinnamate and 4-methylbenzylidene camphor. The most frequently observed UV filters were octyl salicylate and 4-methylbenzylidene camphor, which are among the most prevalent in Australia (O'Malley et al., 2021). Langford and Thomas 2008 found higher UV filter levels during summer in Norway, with octocrylene up to 7,000 ng/L. Overall, UV filter levels in this study are 10–1,000 times lower than other studies (Labille et al., 2020), including one in Australia (O'Malley et al., 2021). This could be due to different sampling approaches, where these studies refer to total concentrations measured in discrete water samples collected at high recreational activity times. Two Swiss studies using SPMD passive samplers found similar levels of octocrylene, octyl methoxycinnamate, and 4-methylbenzylidene camphor in lakes with recreation compared with this study (Balmer et al., 2005; Poiger et al., 2004).

### Seasonal and spatial variations in PAH and UV filter concentrations

Lake Maroon (MAD-SP004) and Lake Moogerah (MOD-SP002) showed significantly higher PAH levels in summer (3.9 ± 1.1 and 4.0 ± 1.2 ng L^−1^ respectively) than winter (1.6 ± 0.61 and 1.5 ± 0.84 ng L^−1^, respectively, Figure 1A; unpaired *t*-test, *p *= 0.015 and *p *= 0.049, respectively), suggesting a relationship between increased boating activity in summer and PAH concentrations. Both sites are popular for high-speed boating, jet skiing, and water skiing, which likely contribute to the elevated summer PAH levels. Lake Maroon (MAD-SP004) and Lake Moogerah (MOD-SP002) also recorded some of the highest UV filter levels in summer, with concentrations of 19 ± 12 ng L^−1^ and 20 ± 4.1 ng L^−1^, respectively (Figure 1B). However, UV filter levels were only significantly higher in summer than winter at Lake Moogerah (MOD-SP002; 20 ± 4.1 ng L^−1^ in summer vs. 4.1 ± 0.01 ng L^−1^ in winter; unpaired *t*-test, *p *= 0.003). This could be due to differences in recreational activities permitted at the two lakes. Lake Moogerah (MOD-SP002) allows swimming, whereas Lake Maroon (MAD-SP004) does not. It is, however, not uncommon for individuals to swim illegally while using petrol-powered boats.

In contrast, other lakes allowing boating displayed more complex patterns, likely influenced by multiple factors. For instance, Lake Somerset (SOD-SP001, SOD-SP010, and SOD-SP011) had varying PAH levels (Figure 1A). In summer, the lowest PAH levels were found at site SOD-SP010 (1.9 ± 0.58 ng L^−1^), compared with the other two stations, because this is located at the north side of the lake and is characterized by a higher riverine influence and reduced speed limit for boating to avoid collision risks. Similarly to Lake Maroon (MAD-SP004), Lake Moogerah (MOD-SP002) and Lake Borumba (BOD-SP001), Lake Somerset is the only other dam that allows jet skiing (in the unrestricted part of the lake), which could explain the higher PAH values found at sites SOD-SP001 (3.3 ± 1.1 ng L^−1^) and SOD-SP011 (2.6 ± 0.83 ng L^−1^) during the summer period (Figure 1A). This pattern was similar for UV filters with lowest levels measured at SOD-SP010 (3.1 ± 0.33 ng L^−1^), which has no designated area to swim (Figure 1B). The location with highest UV filter concentrations (SOD-SP001) is near the Spit, a recreation area with very high visitation and where swimming is allowed. The second highest UV filter concentrations were found at SOD-SP011, which is located at a second recreation area, Kirkleigh, that allows swimming.

Lake Wivenhoe (WID- SP001 and WID-SP004), the largest lake in the study, showed lower PAH concentrations than other boating lakes like Lake Maroon, Lake Moogerah, and Lake Somerset (Figure 1A). This could be attributed to the lake's large volume, diluting contaminants, and its restrictions on boat engine types (only permits four-stroke or high-efficient two-stroke engines) and boating speeds (maximum 6 knots), leading to lower PAH emissions. Despite swimming being permitted at Lake Wivenhoe, both sites at Wivenhoe recorded low levels of UV filters. This could be due to dilution, as both sampling locations are situated away from the designated swimming areas.

For lakes where boating is not allowed, such as Ewen Maddock Dam (EMD-SP001), Lake Advancetown (HID-SP001), and Lake McDonald (LMD-SP001), they recorded higher or similar PAH concentrations in winter compared with summer. This can be attributed to controlled burns during winter, because bushfires are a potential source of PAHs in the aquatic environment (Campos & Abrantes, 2021). From those sites, EMD-SP001 is the only one permitting swimming, resulting in higher levels of UV filters (16 ± 13 ng L^−1^) over summer than the other two (Figure 1B).

**Figure 2.**
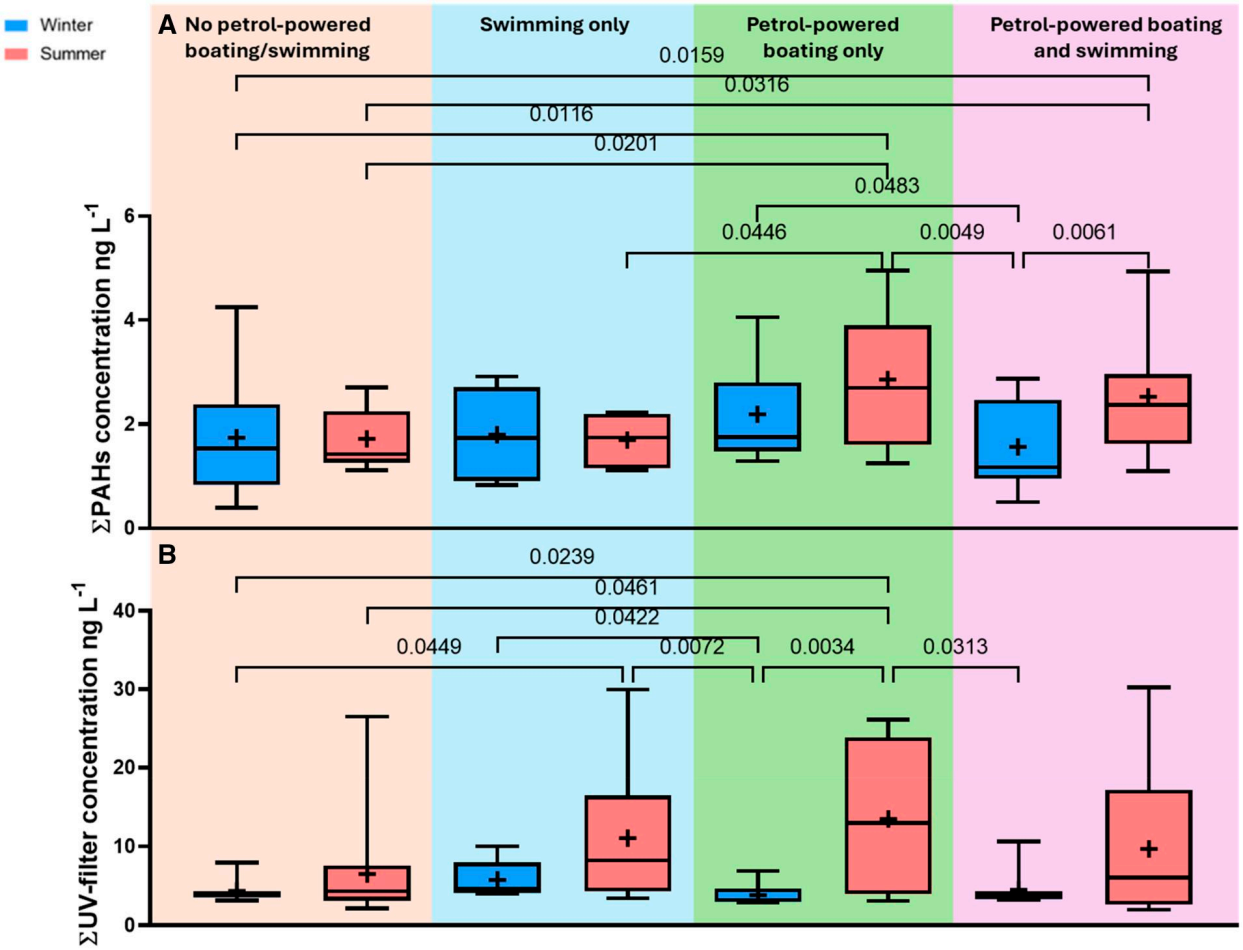
Boxplot of all sites and years combined for polycyclic aromatic hydrocarbons (PAHs, B) and ultraviolet (UV) filter (A) grouped by level of recreation; significant *p* values (Kruskal-Wallis) are shown.

Interestingly, nonboating lakes sometimes showed higher PAH concentrations than boating lakes during summer; similarly, some sites that do not allow swimming had high levels of UV filters. This highlights the multifactorial nature (i.e., lake size, recreational activity type, and seasonal variations) of PAH and UV filters presences. Consequently, we categorized the lakes into four distinct groups based on activity for winter and summer separately: no petrol-powered boating or swimming, swimming only, petrol-powered boating only, and both petrol-powered boating and swimming (Figure 2).

Sites permitting petrol-powered boating showed significantly higher PAH levels in summer compared with all other groups (Kruskal-Wallis test, *p* = 0.01–0.05 Figure 2A), except when compared with their own winter levels and sites allowing both petrol-powered boating and swimming during summer (Figure 2A). This highlights the impact of petrol-powered boating on elevating PAH levels. Additionally, sites that permitted both petrol-powered boating and swimming during summer had significantly higher PAH levels compared with their own winter levels and sites that did not allow petrol-powered boating or swimming (Figure 2A). A possible reason for the similar PAH levels at sites permitting both petrol-powered boating and swimming compared with sites allowing only swimming could be attributed to site-specific factors and the multifactorial nature. The lakes categorized as allowing petrol-powered boating and swimming are among the largest in this study, which may result in greater dilution of PAHs. For UV filter, the pattern was less clear (Figure 2B). A possible reason is that although sites do not allow swimming, they do allow some sort of on-water activity such as petrol-powered boating, or nonpowered/electric boating, and as mentioned above, it is not uncommon for individuals to swim illegally while doing any of the on-water activities.

### Evaluating the toxicity risk: a comparative analysis of PAH and UV filter levels against established guideline values

In Australia, there are guidelines for acceptable levels of PAHs in fresh, marine, and drinking water. The Australian and New Zealand guidelines for fresh and marine water quality were established to provide guidance to authorities on how to manage water quality (Australian and New Zealand Environment and Conservation Council, 2000), in relation to protecting aquatic species, the environment, and for guidance in terms of irrigation, livestock and drinking water. For fresh and marine water, established guideline values (protective of 99% of species) for five PAHs (anthracene, benzo[a]pyrene, fluoranthene, phenanthrene, and naphthalene) range from 0.01 to 50 µg/L for anthracene and naphthalene, respectively. The levels found in this study are below (10–1,000 times) these guideline values. Other studies evaluating different toxicity threshold values set threshold levels at high ng/L–µg/L, much higher than those found in our study (Jesus et al., 2022; Sun et al., 2023; Wu et al., 2011). The Australian drinking water guidelines state that benzo[a]pyrene should not exceed 10 ng/L which is well above the levels found in this study. There is inadequate data to set guideline values for other PAHs. However, comparative carcinogenic potency approximations indicate that the levels found in this study are below any risk thresholds. (National Health and Medical Research Council, 2011). The World Health Organisation has set similar guideline values for PAHs in the aquatic environment (World Health Organisation, 2017). Water quality guidelines for organic compounds are typically expressed in total concentrations. However, when using passive samplers, we measure the “freely available” fraction, which is generally lower than the total concentration. This distinction is crucial, because the freely available fraction represents the portion of the compound that is bioavailable and potentially more relevant for assessing environmental impact and risks. Therefore, it is important to consider how these different measurement approaches might influence the interpretation of water quality data and the subsequent management decisions.

To the best of our knowledge, there are no available water guideline values for organic UV filters. However, a recent review and ecological risk assessment study has generated predicted no-effect concentration values for most UV filters measured in this study. Ultraviolet filter levels observed in this study were at the low ng/L concentration, whereas the established predicted no-effect concentration values are in the µg/L range (Carve et al., 2021). Nevertheless, the continuous discharge of UV filters into our environment, together with their lipophilic nature and bio-accumulative potential, present a significant environmental concern (Gago-Ferrero et al., 2012). Studies have shown that octocrylene, 4-methylbenzylidene camphor, octyl methoxycinnamate, octyl salicylate, and homosalate all have the potential to bioaccumulate in biota (Blüthgen et al., 2014; Buser et al., 2006; Huang et al., 2021; Peng et al., 2017; Zhang et al., 2016). Over time, the concentration of these substances within organisms can reach toxic levels. This accumulation has the potential to act as endocrine disrupting and interfere with reproductive functions (Huang et al., 2021; Kwon & Choi, 2021). Although the passive sampler provides us with a monthly average concentration of UV filters, it is important to consider that these are averages and do not represent acute exposure scenarios. In specific locations, such as those in close proximity to swimming areas, the maximum concentrations might be significantly higher. This increase could potentially result in concentrations that surpass the threshold for acute toxicity (National Academies of Sciences, Engineering, and Medicine, 2022). Therefore, when interpreting the data from passive samplers, it is essential to consider these potential spikes in concentration, especially in areas of high human activity like swimming zones. This will ensure a more accurate and comprehensive understanding of the existing environmental conditions.

Although this study reports on individual UV filter and PAH compounds, the additive effects of a complex mixture of chemicals present in these environments requires consideration in terms of additive toxicity effects (Kortenkamp & Faust, 2018). Therefore, understanding and mitigating the impact of these substances on our environment and the organisms within it is of great importance.

## Conclusion

This study provides a comprehensive analysis of the impact of recreational activities on the presence and concentration of PAHs and UV filters in drinking water reservoirs in Southeast Queensland, Australia. The findings highlight the impact of petrol-powered boating and swimming on water quality with seasonal variations. Chrysene, fluoranthene, and benzo(bjk)fluoranthene were the most frequently detected PAHs. Higher levels of PAHs at sites that allow petrol-powered boating highlights the contribution of these on-water activities to the contamination of water bodies. Ultraviolet filters were generally detected in higher levels in lakes allowing swimming during summer, reflecting the direct release of these chemicals from personal care products used by recreational users. Lakes that do not permit swimming and still present high levels of UV filters may indicate signs of illegal swimming, which is not uncommon during the hot summers in Southeast Queensland, Australia. Despite the detected levels of PAHs being below established guideline values for environmental and human health risks, the persistent discharge and potential bioaccumulation of UV filters may present long-term ecological concern, especially when taken into consideration that passive samplers are only measuring the “freely” available concentration, which is lower than the total concentration to which these guidelines refer. The study emphasizes the need for ongoing monitoring and management strategies to mitigate these risks. The complexity of contaminant presence, influenced by factors such as lake size, type of recreational activity, and seasonal variations, shows it is important to have well-established ongoing water quality management programs. Balancing the social and economic benefits of recreational use of drinking water supplies with the importance of maintaining water quality is crucial. Continued research is essential to develop effective strategies for mitigating the environmental impact of these contaminants and ensuring the sustainability of drinking water resources.

## Supplementary Material

vgaf007_Supplementary_Data

## Data Availability

Data available on request.
