# Supplementary material for: Impact of ultraviolet filters and polycyclic aromatic hydrocarbon from recreational activities on water reservoirs in southeast Queensland Australia
Source: Environ Toxicol Chem. 2025 Jan 9;44(3):674–82. doi: 10.1093/etojnl/vgaf007 (PMC11864206; doi:10.1093/etojnl/vgaf007)
Supplement: vgaf007_Supplementary_Data [file vgaf007_supplementary_data.zip › vgaf007_Supplementary_Data/Supplementary information.docx]

Supplementary information

The Effect of Recreational Activities on the Quality of Drinking Water Lakes in Southeast Queensland, Australia

Rory Verhagen^a*^, Cameron Veal ^b,c^, Elissa O’Malley ^a^, Michael Gallen ^a^, Katrin Sturm ^b^, Michael Bartkow^b^, and Sarit Kaserzon ^a,d^

^a^Queensland Alliance for Environmental Health Sciences (QAEHS), The University of Queensland, 20 Cornwall Street, Woolloongabba, Queensland 4102, Australia.

^b^Seqwater, 117 Brisbane Street, Ipswich 4305, QLD, Australia

^c^School of Civil Engineering, The University of Queensland, St Lucia 4072, QLD, Australia

^d^Queensland Health, Queensland Public Health and Scientific Services Division, 15 Butterfield Street, Herston, QLD 4006, Australia.

Contents

[1. MATERIAL AND METHODS 2](#_Toc162011648)

[1.1 Materials 2](#_Toc162011649)

[1.2 Preparation of Passive Flow Monitors (PFMs) 2](#_Toc162011650)

[1.3 GC-HRMS analysis 3](#_Toc162011651)

# MATERIAL AND METHODS

## Materials

LC-Grade hexane, acetone, dichloromethane, ethyl acetate and methanol were purchased from Merck (Germany). Polytetrafluoroethylene (PTFE) filters (0.22-µm pore size, Agilent Technologies, Australia) were used to filter extracts. Anhydrous sodium sulphate (Sigma-Aldrich, Australia) was used to remove water from sample extracts. Red colour silicone sheeting passive samplers (PDMS) of 0.5 mm thickness were purchased from Powell Industries (Australia, Item no: 99–105913). All glassware was rinsed multiple times with hexane and acetone before use. Isotope labelled PAH standards, d10-fluoranthracene and d12-chrysene (internal standards) were purchased from Cambridge Isotope Laboratories Inc. (America). Solutions of the isotopically labelled PAHs (100 μg/mL) were prepared in hexane/acetone (50:50) and were stored at −20 °C. Sixsteen PAHs including naphthalene (Nap), acenaphthylene (AcNy), fluorene (Fl), acenaphthene (AcNe), phenanthrene (PhA), anthracene (An), fluoranthene (FlA), pyrene (Py), benz[a]anthracene (BaA), chrysene (Chy), benzo[b]fluoranthene (BbF), benzo[k]fluoranthene (BkF), benzo[a]pyrene (BaP), indeno[1,2,3-cd]pyrene (IP), benzo[ghi]perylene (BghiP), dibenz[a,h]anthracene (DBahA) and benzo[e]pyrene (BeP).

Eight UV Filters were purchased from Sigma Aldrich (Australia) and included: (benzophenone-8 (BP-8), homosalate (HMS), 4-methylbenzylidene camphor (4MBC) and 3-benzylidene camphor (3BC)), AccuStandard (benzophenone-3 (BP-3) and octyl methoxycinnamate (EHMC)), Dr Ehrenstorfer (octocrylene (OC) and octyl salicylate (ES)) and Sapphire bioscience (Isoamyl methoxycinnamate (IMC)). Stock solutions of the UV-filters (100 μg/mL) were prepared in methanol and stored at −20 °C.

## Preparation of Passive Flow Monitors (PFMs)

PFMs (in duplicate) were co-deployed with passive samplers and used to estimate water velocity during the deployment period of the samplers (O'Brien et al., 2009; O’Brien et al., 2012). As the rate of diffusion of chemicals into a passive sampling device is a function of the turbulence or water velocity at the surface of the sampler, it is important to monitor this parameter to accurately estimate water concentrations of the target chemicals (Fauvelle et al., 2017; O’Brien et al., 2012). PFMs provide a means of estimating water velocity based on the dissolution of calcium sulphate hemihydrate from the surface of the exposed PFM (13.85 cm^2^).

The PFMs were prepared according to the method of O'Brien et al. (2009) by filling plastic specimen containers (120 mL volume, 42 mm Ø, 105 mm high) with a 1:2 plaster mix prepared using deionised water and dental plaster powder (Boral Australia). Containers were capped once the plaster became firm (after 10 minutes) to prevent drying and were stored at room temperature. The mass of the PFMs was recorded both prior to and after deployment, to determine the mass of plaster lost during deployment.

## GC-HRMS analysis

PAH and UV-filter were analysed on a Thermo Scientific TRACE 1300 gas chromatograph (GC), coupled to a DFS High Resolution mass spectrometer. Thermo Triplus RSH injected 1.6 µL of each sample and was operated in split-less mode with separation achieved on an Agilent J & W DB-5MS column (30 m, 0.25 mm inner diameter, 0.25 mm film thickness of 5% phenyl, 95% PDMS). Experiments were conducted in MID mode at 10,000 resolution (10% valley definition). The inlet, transfer line and source were held at 250°C, 280°C and 280°C, respectively, and the flow rate was maintained at 1.0 mL min^-1^. Similarly, GC ramp rates were used for PAH and UV-filter runs (80°C for 2 minutes; increased to 180°C at 20°C/min and held for 0.5 min; increased to 300°C at 10°C min and held for 8 minutes). The limit of detection (LODs) ranged between the 0.11 and 5.20 ng mL^-1^ for EHMC and 4MBC respectively, calculated as the mean of the lowest concentration at which the signal-to-noise ratio was greater than three, plus 3.3 times the standard deviation. Calibration curves for all chemicals were linear from till 1000 ng mL^-1^ with R^2^ values above 0.99 for each chemical. All data was processed using Xcalibur™ Software - Thermo Fisher Scientific.
